# Fenestrated and Chimney Technique for Juxtarenal Aortic Aneurysm: A Systematic Review and Pooled Data Analysis

**DOI:** 10.1038/srep20497

**Published:** 2016-02-12

**Authors:** Yue Li, Zhongzhou Hu, Chujie Bai, Jie Liu, Tao Zhang, Yangyang Ge, Shaoliang Luan, Wei Guo

**Affiliations:** 1 Department of Vascular Surgery, General Hospital of People’s Liberation Army, Beijing 100853, China; 2 Medical Center Tsinghua University, Beijing, China; 3 Department of Orthopaedic Oncology, Peking University Caner Hospital, Beijing, China; 4 Department of Vascular Surgery, Peking University People’s Hospital, Beijing, China

## Abstract

Juxtarenal aortic aneurysms (JAA) account for approximately 15% of abdominal aortic aneurysms. Fenestrated endovascular aneurysm repair (FEVAR) and chimney endovascular aneurysm repair (CH-EVAR) are both effective methods to treat JAAs, but the comparative effectiveness of these treatment modalities is unclear. We searched the PubMed, Medline, Embase, and Cochrane databases to identify English language articles published between January 2005 and September 2013 on management of JAA with fenestrated and chimney techniques to conduct a systematic review to compare outcomes of patients with juxtarenal aortic aneurysm (JAA) treated with the two techniques. We compared nine F-EVAR cohort studies including 542 JAA patients and 8 CH-EVAR cohorts with 158 JAA patients regarding techniques success rates, 30-day mortality, late mortality, endoleak events and secondary intervention rates. The results of this systematic review indicate that both fenestrated and chimney techniques are attractive options for JAAs treatment with encouraging early and mid-term outcomes.

Endovascular techniques are less invasive methods of treating infrarenal abdominal aortic aneurysms (AAAs)^1^,^2^, especially for patients with severe comorbidities^3^,^4^. However, 30 to 50% of AAA patients are not suitable for elective conventional endovascular repair due to anatomic constraints around the proximal neck^5^,^6^. The term juxtarenal aortic aneurysm (JAA) is routinely used to describe complex AAAs with very short proximal necks. They represent almost 15% of all AAAs^7^,^8^,^9^,^10^,^11^,^12^. These proximal neck adequacy and endograft seal zones have also been identified as key predictors of long term outcomes and success after EVAR^13^,^14^,^15^.

Fenestrated endografts were developed to treat patients with aneurysms with short proximal necks. This technique was first introduced in 1999^16^. Fenestrated grafts extend the proximal sealing zone from the infrarenal segment to the juxtarenal aorta using fenestrations (holes) in the graft or scallops (gaps in the upper graft fabric margin) to permit perfusion of the visceral vessels. This procedure can be performed with or without bridging stents. Greenberg and colleagues first described the use of chimney or snorkel grafts in the endovascular repair of juxtarenal AAA^17^. This procedure involves placement of additional off-the-shelf stents parallel to the main body graft (between the aortic stent and the aortic wall) to facilitate branch vessel perfusion.

Although fenestrated endovascular aneurysm repair (FEVAR) and chimney endovascular aneurysm repair (CH-EVAR) are both effective modalities for treatment of JAAs, their comparative effectiveness is unclear. We conducted a systematic review to compare the outcomes of fenestrated and chimney techniques to traditional methods in the treatment of patients with JAAS. The advantages and limitations of each technique are discussed.

## Methods

### Search strategy

Three independent investigators performed a comprehensive search of PubMed, Medline Embase and the Cochrane Database. The search included all published articles in which patients were diagnosed with JAAAs and treated with F/CH/SN techniques between January 2005 and July 2013. The literature search for relevant articles was performed using the following key words alone and in combination: “juxtarenal aortic aneurysm,” “snorkel grafts,” “chimney graft,” “fenestrated graft,” and “zenith graft.” The search was restricted to articles published in English and human studies. Relevant articles in the reference lists of retrieved articles were searched manually to maximize our search scope. The two authors conducted literature searches independently using the same search terms then discussed which studies fit the inclusion criteria to produce the final list of studies.

### Article selection

The inclusion criteria for this review included original articles reporting more than 5 patients with JAAs treated with F-EVAR or CH/SN-EVAR. Published articles with patients were treated between January 2005 and September 2013. Any studies describing patients treated for endovascular repair of juxtarenal aneurysms were considered. The terms “juxtarenal aneurysm,” “fenestrated,” and “chimney” or “snorkel” were considered sufficient to warrant inclusion. Studies describing elective F-EVAR of juxtarenal aneurysms using any currently licensed stent grafts were included. The basic characteristic of patients, clinical outcomes of complications, graft patency, endoprosthesis-related complications, primary technical success rate, and total mortality were stated. Studies that met any of the follow criteria were excluded: 1) patients treated with a hybrid procedure and multi-branched stent-graft; 2) fewer than 5 patients included; 3) case reports, comments, editorials, review articles, and letters; 4) report of fenestrated or chimney/snorkel technique for pararenal aneurysm repair were excluded.

### Data extraction

Each article was reviewed carefully, and the data were extracted. Patient demographics, including number of patients, gender, mean age (years), aneurysm diameter, aneurysm neck length, date of publication, country of publication, preoperative comorbidity, operative time, fluoroscopy time, contrast dose, estimated blood loss, reconstructed vessels, main stent and fenestrated, chimney/snorkel stent graft involved in the procedural characteristics, were considered. The following clinical outcomes were recorded: success rate, 30-day mortality and cause, over-30-day mortality and cause, patency, duration of follow-up period (months), renal events, endoleak type, number of endoleaks, and major adverse events (MAEs). Data extraction and conversion to the desired format followed the Cochrane Handbook for Systematic Reviews of Interventions guidelines [http://handbook.cochrane.org/ Part 2: General methods for Cochrane reviews >7 studies selected and data collected >7.7 study results extracted and converted to the desired format].

### Statistical analysis

The data are presented as the mean ± standard deviation (SD) or proportions. Comparisons between groups were made using chi-square tests or Fisher’s exact tests for categorical variables. A *P*-value ≤0.05 was considered statistically significant. All analyses were performed using R software version 2.15.1 (http://www.R-project.org/).

## Results

**Figure 1:**
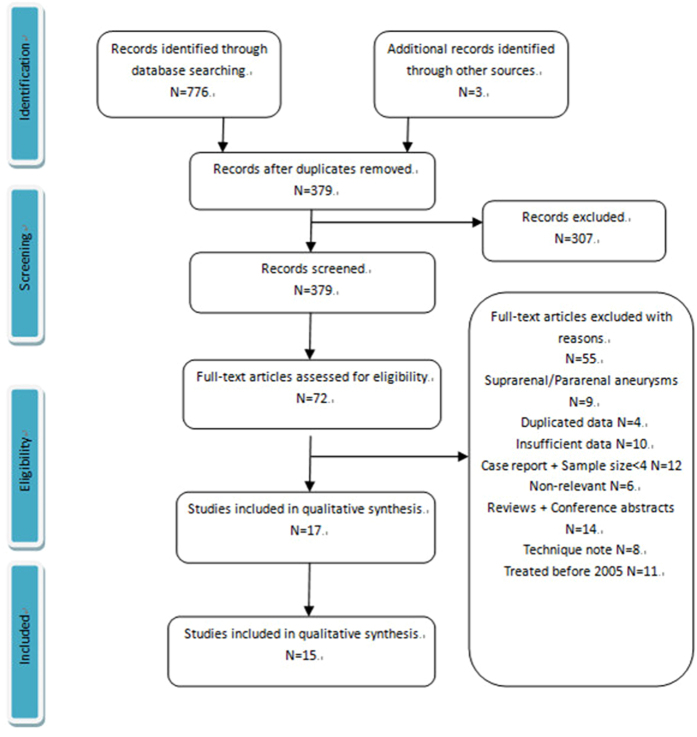
PRISMA flow chart for article selection.

In a total, 776 articles were identified through electronic and manual searching (Fig. 1). Of these, 15 articles met the inclusion criteria and were eligible for analysis, including 8 CH/SN-EVAR case series and 9 F-EVAR case series. Only 2 articles included both CH/SN and F-EVAR cohorts.

### Patient characteristics

All of these patients were treated for juxtarenal aortic aneurysm (JAA) between January 2005 and July 2013. The studies included 700 patients, of whom 542 underwent F-EVAR and 158 underwent CH/SN-EVAR. Patient characteristics are summarized in Tables 1 and 2. The largest series (318 patients) was in the Globalstar study in F-EVAR group. Male patients predominated. In 8 CH/SN-EVAR studies and 9 F-EVAR studies, men comprised 82.9% and 85.7% of the study population, respectively. The mean age was 75 (59–88) years in the CH/SN-EVAR series and 74 (47–86) years in the F-EVAR series. The mean aneurysm diameter was 64.0 mm (47–112) in the F-EVAR group and 64.5 mm (33–110) in the CH/SN-EVAR group. The mean/median aneurysm neck length was 6.7 mm (0–14) in five F-EVAR studies and 2.3 mm (0–10) in six CH/SN-EVAR studies.

All 15 studies reported information on patient co-morbidities (Tables 1 and 2). Nine studies documented smoking status; patients with any history of smoking comprised 69.6% of the patients in the F-EVAR group and 66.1% of those in the CH/SN-EVAR group. Diabetes mellitus was reported in 16.9% and 16.2% of patients in 5 CH/SN-EVAR studies and 6 F-EVAR studies, respectively. The hypertension rates were high in each EVAR group (67% in the F-EVAR group and 87.8% in the CH/SN-EVAR group). Hyperlipidemia was not widely reported in the F-EVAR group. Cardiovascular disease rates were retrieved from all 15 studies. CAD, CHF, MI, arrhythmia, or any combination of these was noted in 33.1% of patients in the F-EVAR series and 44.6% of patients in the CH/SN-EVAR series. In total, 36% and 29.7% of patients in the F-EVAR group and CH//SN-EVAR group, respectively, reported respiratory diseases. Here, 18.6% patients in the F-EVAR series had renal diseases, and 28% of the patients in the CH/SN-EVAR had renal diseases. Previous major abdominal surgery was reported in 4 studies of the CH/SN-EVAR group, which covered a total of 30 patients and 3 studies with 12 patients in the F-EVAR group. Regarding American Society of Anesthesiologists (ASA) grade, the factor was not widely reported, and groups were often combined, making it difficult to extrapolate exact numbers.

### Intra-operative and stent grafts

Here, 7 articles provided procedure time for the CH/SN-EVAR group, which averaged 178 (75–810) minutes. In addition, 6 studies documented a mean of 54.6 (15–290) minutes of fluoroscopy time. According to 7 articles, the median volume of contrast used was 146 (45–465) ml. Blood loss was reported in 5 series. The estimated mean volume was 332 ml and ranged from 30 to 2204 ml Table 3. A great variety of aortic stent grafts were utilized as the main body in all 7 studies (121 patients 76.6%). The main aortic stent grafts implanted included the Zenith (Cook Inc., Bloomington, IN, U.S.) (53 patients, 43.8%); Endurant (Medtronic Inc., Minneapolis, MN, U.S.) (40 patients, 33.1%); Excluder (W. L. Gore and Associates, Newark, DE, U.S.) (14 patients, 11.6%); Renu (Cook Inc., Bloomington, IN, U.S.); TX2 (Cook Inc., Bloomington, IN, U.S.); Powerlink (Endologix Inc., Irvine, CA, U.S.); Talent (Medtronic Inc., Minneapolis, MN); TAG (W. L. Gore and Associates, Newark, DE, U.S.); and Trivascular Ovation (Ovation; TriVascular Inc., Santa Rosa, CA, U.S.) (Table 4). A total of 229 visceral vessels were treated with stents. An additional 7 studies listed the specific stent type (121 patients, 76.6%). The most commonly used chimney grafts included Advanta (Atrium Medical Corporation, Hudson, NH U.S.) (47, 26.6%); iCAST stents (Atrium Medical Corporation, Hudson, NH, U.S.) (46, 26%); Viabahn (W. L. Gore and Associates, Newark, DE, U.S.) (39, 22%); Lifestent (C.R. Bard, Murray Hill, NJ, U.S.); and Luminexx and Fluency stents (both from C.R. Bard, Murray Hill, NJ, U.S.) (7.34% each) (Table 4).

Seven articles of the F-EVAR group reported a mean procedure time of 261 (80–554) minutes. Six studies documented the mean volume of contrast. The average volume was 166, ranging from 90 to 465 ml. The mean duration of fluoroscopy was 64 (5–223) minutes, and estimates of blood loss ranged from 50 to 7000 ml (mean 534 ml) (Table 5). Custom-made Zenith grafts (Cook Medical) were the most widely implanted as the main graft (517 patients, 95.3%). In the study reported by Dijkstra, 25 patients received Anaconda stents (Vascutek, Renfrewshire, Scotland, U.K.) as the main body graft (4.7%). A total of 1082 visceral vessels described in 9 articles were implanted with stent grafts. Advanta (Atrium Medical Corporation, Hudson, NH U.S.) was the most commonly used visceral stent (603, 55.7%) followed by Zenith (Cook Inc., Bloomington, IN, U.S.) (58, 5.4%); iCAST (Atrium Medical Corporation, Hudson, NH U.S.) (53, 4.9%); Plamaz (Cordis Corporation, Johnson & Johnson Company, Miami, FL, U.S.) (49 vessels, 4.5%); EV3 (ev3Endovascular Inc., Plymouth, MN, U.S.) (Table 6). In addition, the stent type used (cover or bare, balloon or self-expansion) was not specified for 134 visceral vessels. The technical success rate of the chimney operation was 97.4% and 98.8% for fenestrated operations.

### Postoperative

The length of the hospital stay was reported for the F-EVAR group in 7 studies (mean, 7 (1–100) days). This information was not widely reported for the CH/SN group. Only 3 articles provided this information. The average length of hospital stay was 4.4 (2–50) days, which is likely an underrepresentation. The patency rate was 95.9% in the F-EVAR group and 97% in the CH-EVAR group (Tables 7 and 8).

Post-operative major adverse events (MAE) of F-EVAR included 42 cardiac events (7.7%), 25 respiratory events (4.6%), 12 gastrointestinal events (2.2%), 11 neurological problems (2%), and 11 ischemic problem (2%). Here, 5 patients suffered from spinal ischemia^18^. One patient eventually made a full recovery, two made a partial recovery, and two did not recover. Cardiac events were also commonly reported after chimney/snorkel operations as noted in 4.5% of cases (5 of 110 patients from 6 studies). The wound complication rate was 6.36%.

The 30-day mortality rate was 3.8% (6/158) in the CH/SN-EVAR group and 1.1% (6/542) in the F-EVAR group. Six deaths occurred after CH/SN-EVAR, including three cases of MOF (multiple organ failure) caused by bowel ischemia and three cardiac events with no intraoperative deaths. One patient in the fenestrated group died due to myocardial infarction (MI), one died from pneumonia, one died of multisystem organ failure, and one patient died of bowel ischemia related to AAA. The reasons for the last two deaths were not specifically listed but were not related to AAA.

The mean follow-up was 14.7 months in the CH/SN-EVAR group (range 0–46 months) and 12.8 months in the F-EVAR group (range 1–65 months). During follow-up, 15 (8.46%) deaths occurred in the CH/SN-EVAR group (range 1–26 months) In addition, 29 patients (5.4%) died in the fenestrated group, and the last death occurred in the 51th month postoperation. After the chimney/snorkel operation, 7 (7/15 46.6%) patients died of cardiac causes. Two patients died of AAA rupture, and 4 died for reasons that were not specified but not related to AAA^19^. The 29 deaths that occurred after the fenestrated operation, including 4 deaths caused by cardiac and respiratory factors, 3 deaths due to MOF, 3 deaths due to stroke, 1 death due to CKD, 1 death due to gastrointestinal bleeding, 1 death due to tumor, and 16 deaths with non-specific causes but not related to AAA. A total of 21 deaths (13.3%) occurred in the CH/SN group, and 35 deaths (6.4%) occurred in the F-EVAR group. Cardiac disease was the most common cause of postoperative death (reported death cause).

Secondary interventions included the resolution of endoleaks, target vessel occlusion or stenosis, limb occlusion or stenosis, and excess bleeding or hematoma. A total of 58 secondary intervention events occurred after fenestrated operation, 15 of which were noted in the CH/SN-EVAR group.

**Figure 2:**
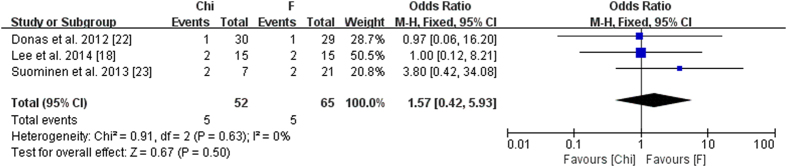
Meta-analysis on two-arm studies.

The comparison results were extracted from two groups of studies and provided in Table 5. We also performed a meta-analysis on two-arm studies (Fig. 2).

### Endoleak events

The endoleaks were divided into groups according to leak site as described by Veith *et al*.^20^: type I, attachment site leak; type II, branch leak; type III, graft defect; and type IV, graft wall (fabric) porosity. Pooled analyses were performed by calculating the overall rates of events. All 15 articles reported endoleak events. Among a total of 106 patients, 19.6% of patients in the F-EVAR group (106/542) and 14.6% of patients in the CH/SN-EVAR group (29/158) experienced endoleak events. In the F-EVAR series, 29 type I (5.35%, 29/542), 69 type II (12.7%, 69/542), and 13 type III (2.4%, 13/542) endoleaks were detected. Of these, 9 type I endoleaks and 5 type III endoleaks were diagnosed intraoperatively and treated successfully using the kissing balloon technique, Palmaz stents, proximal coil embolization, or extender cuff placement. In addition, 4 type I, 9 type II, and 5 type III endoleaks required secondary intervention to seal the leak after the primary procedure. However, 2 type II endoleaks were not resolved by this treatment. These 2 patients required further observation. A total of 18 endoleaks disappeared during follow-up without any treatment. Here, 12 type I, 12 type II, and 12 type III became stable, making treatment unnecessary. No sac enlargement, complications, or deaths were observed during follow-up.

Post-procedural CT scans showed 12 type I (7.6%, 12/158), 16 type II (10.1%, 16/158) and 1 type III endoleaks in the CH/SN-EVAR group. Here, 6 patients (2 type I, 4 type II) required secondary intervention to resolve the endoleak. A total of 12 endoleaks resolved spontaneously during follow-up, ranging from 1 to 12 months. In addition, 7 type II endoleaks and one type I endoleak were under surveillance during follow-up. According to Schiro, two deaths were caused by aneurysm rupture related to a type I endoleak^19^.

### Renal events

Here, 49 renal artery events were reported in F-EVAR group, including stenosis (26), occlusions (12), perforation/bleeding (7) and stent events (4). Of these, 5 events were resolved during the operation, and 32 events required secondary intervention. Four cases of renal artery occlusion were documented in the CH/SN-EVAR group.

Of the 15 studies included in the review, 10 reported renal events. These events were defined as an increase in serum creatinine to >2 mg/dl or by >30% relative to baseline during the peri-operative period. Of the 542 relevant patients, 30 (5.5%) developed renal impairment or failure following F-EVAR. In addition, 16 suffered from postoperative renal impairment. This complication was temporary for 7 patients, and only 2 patients required temporary or persistent postoperative dialysis. In contrast, 31 patients (19.6%) in the chimney group developed this complication, and 1 patient required persistent dialysis.

## Discussion

The present review compared the clinical outcomes of patients who underwent F-EVAR and the chimney/snorkel technique for treatment of juxtarenal aortic aneurysms. The fenestrated technique exhibited advantages compared with the chimney/snorkel technique with respect to 30-day mortality, late mortality and renal adverse events. However, patients in the CH/SN-EVAR group experienced shorter operative and fluoroscopy procedures, required lower contrast doses, and suffered less blood loss during the operation. The present study aimed to evaluate the safety and efficacy of fenestrated and chimney techniques for JAAA.

The fundamental goal of fenestrated and chimney/snorkel techniques is to extend the sealed area and maintain flow to a branch vessel with or without the use of a stent-graft. F-EVAR is an expensive procedure that is tailored specifically to each individual patient’s anatomy. The design of each fenestrated device is very complicated and requires accurate calculations of the distances between the visceral vessels. This procedure can easily take 4 to 6 weeks or more in centers lacking staff experienced in this method, where measurements must be double-checked. This technique is costly, time-consuming and not suitable for urgent situations^21^,^22^,^23^,^24^. The chimney/snorkel technique is widely available and can be performed in smaller centers. The technique is less complex and can be performed with off-the-shelf endografts. The technique can be used to provide immediate treatment in acute cases^25^. The major concerns regarding chimney/snorkel are endoleaks and subsequent complications^25^,^26^.

With larger delivery systems, fenestrated grafts must use conduits to open the arteries to insert the transfer system. This procedure may increase the mean operative time and blood loss relative to chimney techniques (operative time: 261 min for F-EVAR vs 178 min for CH-EVAR; estimated blood loss: 534 ml for F-EVAR vs 332 ml for CH-EVAR). Fluoroscopy time and contrast dose were both slightly increased in the fenestrated series compared with the chimney series (64 min vs 54.6 min for fluoroscopy; 166 ml vs 146 ml for contrast dose). This finding is potentially attributed to the fact that the graft can be placed more accurately, and secondary procedures are often performed to verify that the new position is suitable.

Not surprisingly, 30-day mortality rates favor F-EVAR over CH/SN-EVAR (1% vs 3.8%) (Table 9). The increased 30-day mortality rate of CH/SN-EVAR may be attributed to the inclusion of acute patients (acute or semi-acute) and patients with more challenging anatomical structures. Late mortality was 5.35% in the F-EVAR group and 9.5% in the CH/SN-EVAR group. The all-cause death rate was 6.46% (35 patients) in the F-EVAR series and 13.3% (21 patients) in CH/SN-EVAR series. One possible explanation for the relatively increased mortality in the CH group is postprocedural renal dysfunction, which is a strong indicator of poor long-term survival^27^. In the current review, 30 (5.5%) renal events (renal impairment or failure) were reported in the F-EVAR series; 21.5% (34/158) patients suffered from postprocedural renal impairment or failure. Age is also a well-known predictor of mortality after AAA repair.

Endoleak is the most common procedure-specific feature and complications of chimney/snorkel and fenestrated grafting. The postoperative rate of type I endoleak was 7.6% (12/158) in the CH/SN-EVAR group, which was increased compared with the F-EVAR group (3.7% (20/542)) in the current review, excluding nine endoleaks of F-EVAR and one endoleak of CH/SN-EVAR that was detected and treated intraoperatively. In contrast to F-EVAR, chimney grafts were positioned along the outside of the main abdominal endograft and rely on the close conformation of the endograft and the aortic wall around the chimney stent. The gaps that formed between the grafts and the aortic wall can be imagined as small cylinders and conduits (CGs and main graft) within a larger cylinder (the aorta). The gaps may have increased the risk of type I endoleakage in the CH-EVAR group. Oversizing was considered an effective method of narrowing the gaps. In this study series, 5 studies, including 2 F-EVAR studies, reported increases in the main stent size ranging from 10% to 30%. Lachat proposed an elliptical model for the estimation of the appropriate aortic stent graft diameter. Generally, to facilitate the formation of a good seal, the graft should increase in size by 30%. Some authors also recommend that the endograft should be up to 40% oversized to minimize the effects of the chimney gaps^28^. The ideal amount of oversizing remains undetermined^29^. Recent *in vitro* data demonstrated that increasing oversizing significantly decreased the sizes of gap areas, but main endograft in-folding was also detected in most oversized stentgrafts^28^. Interestingly, 8 type I endoleaks disappeared during follow-up. We hypothesize that the longer the gutters, the more resistance to blood flow and the more likely the gutters will thrombose. However, no evidence is provided to support these hypotheses. A high secondary intervention rate was noted after F-EVAR and CH/SN-EVAR. The reintervention rate was approximately 10.7% in the F-EVAR group and 9.95% of in the CH/SN-EVAR group during follow-up. Persistent endoleakage, renal artery stenosis, occlusion, and bleeding all require secondary intervention to relieve these procedures.

One limitation of this study is that some studies did not report all relevant information (i.e., the aneurysm neck length, information regarding stents, fluoroscopy time, and blood loss are not widely reported). Second, case studies and technical reports were excluded. The small number of patients included was insufficient for analysis, and this limitation may have led to underestimation of the rate of post-procedural complications. A number of acute and semi-acute procedures were performed in the CH/SN-EVAR group, whereas fenestrated stents required 4 to 6 weeks to measure and manufacture. Publication bias must also be acknowledged. Nevertheless, this review describes the current state of experience with fenestrated and chimney/snorkel techniques and provides considerable insight into the potential indications, technical considerations, and complications associated with these procedures. Juxtarenal aneurysm has no standard classification system that was applied throughout the current published works on EVAR; however, each of these reports cases of JAA. JAA was defined as cases in which the cross-clamp could not be placed above the infrarenal area safely during open surgery. In studies of EVAR, the term JAA typically refers to normal inter-renal aortic aneurysms without renal artery involvement. There are two situations in which it is unclear whether the term JAA should be applies: 1) extension of the AAA immediately above the inter-renal aorta and 2) aneurismal involvement of renal artery origins with an otherwise normal inter-renal aorta^30^. True comparisons of F-EVAR and CH/SN-EVAR can be made only when study participants are anatomically homogeneous. In the endovascular era, any new classification of JRA should include the location and diameter of the aneurysm and the length and angulation of the aneurysm neck.

## Conclusion

F-EVAR and CH-EVAR techniques are both effective treatment for JAAs patients. The fenestrated technique was considered the priority treatment for JAAs, whereas CH-EVAR is frequently performed in patients with more complex anatomy and urgent cases. Although the early and mid-term outcomes are satisfactory, the long-term durability of these techniques requires further assessment.

## Additional Information

**How to cite this article**: Li, Y. *et al*. Fenestrated and Chimney Technique for Juxtarenal Aortic Aneurysm: A Systematic Review and Pooled Data Analysis. *Sci. Rep*. **6**, 20497; doi: 10.1038/srep20497 (2016).

## Figures and Tables

**Table 1:** Patient demographics of all chimney/snorkel endovascular aneurysm repair (CH/SN-EVAR) case series.

References	Type of study	Patients	Sex (M/F)	Age	Major Comorbidities	Aneurysm diameter (mm)	Length of aneurysm neck (mm)	Treatment period	Country
Donas *et al*. 2012^31^	2 arms	30	27:3	74.8 ± 7.3	22 CAD; 7 RI; 10 respiratory disease;	62	N.D.	2008.1–2010.12	Germany
					10 MI; 11 previous aortic intervention;				
					10 previous aortocoronary bypass or intervention;				
Suominen *et al*. 2013^32^	Single arm	7	5:2	79	1 DM; 1 Hyperlipidemia; 4 HTN; 6 CAD; 3 respiratory; 6 renal failure; 2 smokers;	65 ± 7	2.5 (0-10)	2007.12–2011.8	Finland
Lee *et al*. 2014^33^	2 arms	43	30:13	75(59–88)	40 smoker; 43 HTN; 41 Hyperlipidemia; 30 CAD; 13 CHF; 18COPD; 5 DM;	66 ± 11.9 (51–105)	1.6 ± 2	2009–2012	U.S.
					10 prior AAA repair				
Schiro *et al*. 2013^19^	Single arm	9	6:3	77(65-88)	8 HTN; 2 DM; 5 CAD; 6 Hyperlipidemia; 4 COPD; 5 smokers; 3 RI; 3 CVD;	73 (58–110)	N.D.	2008.7–2012.2	U.K.
Ducasse *et al*. 2013^34^	Single arm	22	21:1	73(63-88)	17 CAD; 6MI; 3 CHF; 4 ejection fraction;	58.5 (45–100)	4.5 (1-9)	2010.7–2012.11	Multiple Center
					13 previous interventions; 18 HTN;				
					17 hyperlipidaemia; 26 smoker; 4 COPD; 2 DM; 4 RI; 4 hostile abdomen; 8 PAD;				
					2 CVD;				
Tolenaar *et al*. 2013^35^	Single arm	5	4:1	75.9(68-85)	3 MI; 3 COPD; 1 ICD 2 arrhythmia; 1 RI;	64.6 (54–72)	4 (0-7)	2009.10–2011.7	Netherland
Lgari *et al*. 2014^36^	Single arm	5	4:1	78.4(76-84)	5 HTN; 4 COPD; 2 CHD; 2 CVD; 1 CAD; 1 Hostile abdomen; 2 malignant disease	60 (33–85)	5.7 (3–10)	2010.1–2013.7	Tokyo
Banno *et al*. 2014^37^	Single arm	37	34:3	74.3 ± 8.7	15 CAD; 14 CHF; 10 Arrhythmia; 9 RI;	65.9 ± 15.3	2.3 ± 3.1	2006.1–2013.4	France
					11 COPD; 30 HTN; 22 Hyperlipidemia;				
					10 DM; 4 CVD; 11 PAD; 1 dialysis;				
					9 prior aortic surgery 5 smokers;				

**N.D.** not documented; **CAD**, coronary artery disease; **RI**, renal impairment; **MI**, myocardial infarction arrhythmia; **DM**, diabetes mellitus; **HTN**, hypertension; **CHF**, congestive heart failure; **PAD**, peripheral arterial disease; **CVD**, cerebrovascular disease; **CRF**, Chronic renal failure; **CKD**, Chronic kidney disease; **ICD,** implantable cardioverter defibrillator;

**Table 2:** Patient demographics of all Fenestrated endovascular aneurysm repair (F-EVAR) case series included.

References	Type of study	Patients	Sex (M/F)	Age	Major Comorbidities	Aneurysm diameter (mm)	Length of aneurysm neck (mm)	Treatment period	Country
Lee *et al*. 2014^38^	Single arm	15	10:5	77.4	11 smokers; 12 CAD; 7 CHF; 14 HTN;	61.6 (47–105)	4.5 (2–8)	2012–2013	U.S.
					2 COPD; 3 DM; 1 Prior AAA repair;				
Globalstar. 2012^18^	Single arm	318	274:44	74 (47–86)	44 DM; 196 HTN; 149 CAD; 19 CHF;	65 (46–112)	N.D.	2007.1–2010.12	U.K.
					44 Renal Failure; 27 CVD;				
					3 Previous aortic surgery;				
Liao *et al*. 2014^39^	Single arms	8	4:4	75 (64–85)	3 CAD; 5 COPD	58 (52–63)	6 (4–12)	2012.8–2013.5	U.S.
Dijkstra *et al*. 2014^40^	Single arm	25	22:3	73 ± 7.1	3 DM; 15 HTN; 9 Hyperlipidemia;	61 (55–88)	N.D.	2011.5–2013.9	Duth
					9 smoker; 18 cardiac; 8 renal disease;				
					3 pulmonary disease;				
Donas *et al*. 2012^31^	2 arms	29	29:0	73.7 ± 6.1	24 cardiac; 5 RI; 11 respiratory; 7 MI;	65	N.D.	2008.1–2010.12	Germany
					8 previous aortic intervention;				
					12 aortocoronary surgery;				
Suominen *et al*. 2013^32^	2 arms	21	21:0	73	4 DM; 9 hyperlipidemia; 13 HTN; 14 CAD; 6 respiratory disease; 19 renal failure;	65 ± 7	2.5 (0–2.5)	2007.12–2011.8	Finland
					4 smokers;				
Greenberg *et al*. 2009^41^	Single arm	30	24:6	75 (59–86)	8 MI; 3 CHF; 15 CAD; 14 arrhythmia;	61.4 ± 9.7 (48.8–88)	9.2 ± 2.9 (2.4–14.4)	2005.1–2006.1	Global
					26 HTN; 5 thromboembolic event; 7 PAD;				
					9 COPD; 2 RI; 7 DM; 5 CVD; 27 smokers;				
Tambyraja *et al*. 2011^42^	Single arm	29	27:2	74 (54–86)	21 smoker/COPD; 15 HTN; 13 MI; 3 CRF ;	68 ± 7	N.D.	2005.10–2010.3	U.K.
					4 hostile abdomen;;2 CVD; 3 cardic failure				
Oderich *et al*. 2014^43^	Single arm	67	54:13	74 ± 8	60 HTN; 59 smokers; 36 CAD; 20 MI;	60 ± 10 (47–100)	7.5 ± 2.3 (4–12)	2005–2012	U.S.
					21 Arrhythmia; 24 COPD; 16 CKD; 16 DM; 15 PAD; 11 CVD; 7 CHF;				
					7 history of thromboembolic event;				

**N.D.** not documented; **CAD**, coronary artery disease; **RI**, renal impairment; **MI**, myocardial infarction arrhythmia; **DM**, diabetes mellitus; **HTN**, hypertension; **CHF**, congestive heart failure; **PAD**, peripheral arterial disease; **CVD**, cerebrovascular disease; **CRF**, Chronic renal failure; **CKD**, Chronic kidney disease.

**Table 3:** Procedural characteristics of CH/SN-EVAR cohort.

References	Operative time (min)	Fluoroscopy time (min)	Contrast dose (ml)	Estimated Blood loss (ml)	Technique success rate
Donas *et al*. 2012^31^	89 ± 21	44.8 ± 13.2	112 ± 23	N.D.	97.70%
Suominen *et al*. 2013^32^	213 ± 67 (118–351)	71 (43–189)	267 ± 80 (120–465)	425 (100–2200)	93%
Lee *et al*. 2014^33^	237 (110–810)	77.8 ± 48.1 (30–290)	180.5 ± 66.2(66–400)	428 (100–2000)	N.D.
Schiro *et al*. 2013^19^	187 ± 30	41 ± 11	194 ± 52	212 ± 102	N.D.
Ducasse *et al*. 2013^34^	105 (75–290)	23 (15–55)	65 (45–120)	55 (30–550)	100%
Tolenaar *et al*. 2013^35^	N.D.	N.D.	N.D.	N.D.	92.3%
Lgari *et al*. 2014^36^	171 (107–511)	N.D.	105 (100–200)	235 (100–2204)	100%
Banno *et al*. 2014^37^	183 ± 69	43	139 ± 102	N.D.	N.D.

**N.D.** not documented.

**Table 4:** Data on aortic stent grafts and chimney/snorkel stent graft utility.

References	Stented vessels: RRA/LRA/SMA/CA	Main stent	Chimney/snorkel stent grafts
Donas *et al*. 2012^31^	Stented vessels: 38	30 Endurant stent graft^e2^	*Covered balloon expandable*
	RRA/LRA/SMA		38 Advanta^c^
	19/16/3		
Suominen *et al*. 2013^32^	Stented vessels: 9	7 Excluder^a1^	9 Advanta^c^
	3 RRA/6 LRA		
Lee *et al*. 2014^33^	Stented vessels: 74 RA	27 Zenith bifurcated EVAR system^b1^	*Balloon-expandable*
		6 Endurant^e2^	46 iCAST covered stents^c^ (5, 6, or 7 mm _59 mm)
		1 Talent^e2^	*Self-expanding*
		5 Renu^b1^	27 Viabahn covered stents^a2^
		2 TX2^b1^	(5, 6, or 7 mm _ 50 mm)
		1 TAG^a1^	*Bare stent*
		2 Excluder^a1^	1 Omnilink Elite^i^
Schiro *et al*. 2013^19^	Stented vessels: 9	6 Zenith^b1^	9 Fluency^d1^
		1PowerlinkEndologix^g^	3 Luminexx^d2^
		1 Talent^e1^	
		1 Trivascular Ovation^l^	
Ducasse *et al*. 2013^34^	Stented vessels: 22	12 Zenith LP^b1^	13 Lifestent^d1^
		6 Zenith Flex^b1^	3 Absolute^i^
		2 Zenith AUI^b1^	2 Astron^j^
		1 Endurant^e1^	2 Epic^f1^
		1 Powerlink^g^	1 S.M.A.R.T^h1^
			1 Everflex^k1^
Tolenaar *et al*. 2013^35^	Stented vessels: 8	3 Endurant^e1^	12 Viabahn^a1^
	RRA/LRA/SMA	2 Excluder^a1^	1 Fluency^d1^
	4/4/0		
Lgari *et al*. 2014^36^	Stented vessels: 9	3 Excluder^a1^	7 Express SD^f2^
	4 RRA/5 LRA	2 Endologic Powerlink bifurcated graft^g^	1 Coyote
			1 SHIDEN
Banno *et al*. 2014^37^	Stented vessels: 60	N.D.	N.D.
	RRA/LRA/SMA:		
	24/26/10		

a1. W. L. Gore and Associates, Newark, DE, U.S. a2. W. L. Gore, Flagstaff, AZ, U.S. b1 .Cook Inc, Bloomington, IN. U.S. b2. Cook Australia Ltd., Australia. b3. William A. Cook Australia, Ltd., Brisbane, Australia b4. Cook Medical, Canvey Island, U.K. c Atrium Medical Corporation, Hudson, NH, U.S. d1. C.R. Bard, Murray Hill, NJ, U.S. d2. Bard Peripheral Vascular, Inc. e1. Medtronic, Inc, Minneapolis, MN, U.S. e2. Medtronic Vascular, Santa Rosa, CA, U.S. f1. Boston Scientific, Natick, MA, U.S. f2. Boston Scientific, Bloomington, MN, U.S. g Endologix, Inc, Irvine, CA,U.S. h1 Cordis Corporation, Johnson & Johnson Company, Miami, FL, U.S. h2. Cordis, Warren, NJ, U.S. i Abbott Vascular, Temecula, CA, U.S. j Biltronic, Bulach, Switzerland. k1 ev3Endovascular Inc, Plymouth, MN. U.S. k2. Covidien, Plymouth, CA, U.S. l Ovation; TriVascular Inc., Santa Rosa, CA, U.S > m. Vascutek, Renfrewshire, Scotland, U.K.

**Table 5:** F-EVAR procedural characteristics of F-EVAR cohort.

References	Operative time (min)	Fluoroscopy time (min)	Contrast dose (ml)	Estimated blood loss (ml)	Technique success rate
Lee *et al*. 2014^38^	282	99	123.04	650	96%
Globalstar. 2012^18^	271 (80–720)	N.D.	N.D.	807 ± 500 (50–7000)	99%
Liao *et al*. 2014^39^	N.D.	55 (17–85)	90 (42–122)	N.D.	100%
Dijkstra *et al*. 2014^40^	240 (190–356)	67 (53–107)	194 (103–320)	N.D.	94.6%
Donas *et al*. 2012^31^	290 ± 122	54.3 ± 12.2	156 ± 56	N.D.	N.D.
Suominen *et al*. 2013^32^	213 ± 67 (118–351)	71 (43–189)	267 ± 80 (120–465)	425 (100–2200)	93%
Greenberg *et al*. 2009^41^	234 (170–554)	N.D.	N.D.	601 (50–2400)	100%
Tambyraja *et al*. 2011^42^	N.D.	N.D.	N.D.	200 (50–3000)	100%
Oderich *et al*. 2014^43^	236 ± 81 (104–554)	60 ± 34 (5–223)	N.D.	526 (50–2400)	100%

**N.D.** not documented.

**Table 6:** Data on aortic stent grafts and fenestrated stent graft utility.

References	Stented vessels: RRA/LRA/SMA/CA	Main Stents	Fenestrated stent grafts
Lee *et al*. 2014^38^	Stented vessels: 25	15 ZFEN^b1^	*Covered stents:*
			25 iCAST^c^
Globalstar 2012^18^	Target vessels: 889	318 Zenith^b4^	*Bare metal stents: 63 vessels*
	Stented vessels: 670.		35 Palmaz Genesis^h1^.
	RRA/LRA/SMA/CA		13 EV3^**k**^
	269/278/113/10		7 Luminexx^d2^
			2 AVE^e1^
			1Express^f1^
			5 unspecified bare stents
			*Covered stents :529 vessels*
			522 Advanta^c^
			4 Jostent^i^
			3 Fluency^d2^
			78 unspecified covered stents.
Liao *et al*. 2014^39^	Target vessels: 21	8 Zenith^b1^	*Covered balloon-expandable stents:*
	Stented vessels: 8		8 iCAST^c^
Dijkstra *et al*. 2014^40^	Stented vessels: 56	25 Anaconda^m^	*Covered stents for all renal artery*
			54 Advanta V12^c^
			2 unspecified bare stents
Donas *et al*. 2012^31^	Stented vessels: 44	29 Zenith^b1^	*Covered balloon expandable*
			32 Advanta^c^
			*Bare balloon expandable*
			12 Palmaz^h1^
Suominen *et al*. 2013^32^	Target vessels: 54	21 Zenith^b2^	*Covered stents :*
	Stented vessels: 49		49 Advanta V12^c^
	RRA/LRA/SMA		
	17/16/21		
Greenberg *et al*. 2009^41^	Target vessels: 77	30 Zenith^b1^	N.D.
	Stented vessels: 54		
Tambyraja *et al*. 2011^42^	Target vessels: 79	29 Cook Zenith^b3^	29 unspecified covered stent
			18 unspecified bare stent
			2 unspecified stent
	Stented vessels: 49		
Oderich *et al*. 2014^43^	Target vessels: 178	67 Zenith^b1^	58 Zenith alignment stent^b1^
	Stented vessels: 127		
			29 Express LD stent^f1^
			25 eV3 IntraTherapeutics stent^k2^
			20 iCAST Covered stent^c^
			2 Palmaz Genesis stent^h2^
			1 Bridge Assurant stent ^e1^

a1. W. L. Gore and Associates, Newark, DE, U.S. a2. W. L. Gore, Flagstaff, AZ, U.S. b1 .Cook Inc, Bloomington, IN, U.S. b2. Cook Australia Ltd, Australia. b3. William A. Cook Australia, Ltd., Brisbane, Australia b4. Cook Medical, Canvey Island, U.K. c Atrium Medical Corporation, Hudson, NH, U.S. d1. C.R. Bard, Murray Hill, NJ, U.S. d2. Bard Peripheral Vascular, Inc.US e1. Medtronic, Inc, Minneapolis, MN, U.S. e2. Medtronic Vascular, Santa Rosa, CA, U.S. f1. Boston Scientific, Natick, MA, U.S. f2. Boston Scientific, Bloomington, MN, U.S. g Endologix, Inc, Irvine, CA,U.S. h1 Cordis Corporation, Johnson & Johnson Company, Miami, FL, U.S. h2. Cordis, Warren, NJ, U.S. i Abbott Vascular, Temecula, CA, U.S. j Biltronic, Bulach, Switzerland. k1 ev3Endovascular Inc, Plymouth, MN, U.S. k2. Covidien, Plymouth, CA, U.S. l Ovation; TriVascular Inc., Santa Rosa, CA, U.S. m. Vascutek, Renfrewshire, Scotland, U.K.

**Table 7:** CH/SN-EVAR cohort clinical outcome.

Authors	MAE (major adverse events)	30-day mortality	Cause of death	Over 30day mortality	Cause of death	Patency (6 months)	Follow-up (months)	Length of stay (days)	Secondary intervention rate
Donas *et al*. 2012^31^	1 MI; 2 Type II endoleaks; 1 RA occlusion;	0		N.D.	N.D.	97.4%	15.2 ± 6.2	3.5	3.3%
Suominen *et al*. 2013^32^	1 MI; 4 wound Infection; 1 common ilac artery embolism; 1 Type II endoleak; 2 RFI;1 Renal stent twist	0		3	2 M.I. (5 and 7 months) 1 lower limb ischemia	N.D.	22 (1–46)	N.D.	25%
Lee *et al*. 2014^33^	3 Type I, 6 Type IIand 1 Type III endoleaks; 19 RFI;	2	2 M.I.	4	4 M.I.	95% (24 months)	21.1 (2.6–40.4)	N.D.	4.7%
Schiro *et al*. 2013^19^	1 MI; 1 arrhythmia; 5Type I endoleaks; 1 ARF(need dialysis)	0		2	2 AAA rupture (11 and 16 months, caused by type I endoleak)	N.D.	12 (5–24)	N.D.	0
Ducasse *et al*.2013^34^	1 stroke; 1 lower limb embolism; 1Type I; 4Type IIendoleaks; 2 ARF;2 accessory renal artery occlusion	1	1 acute heart disease	0		N.D.	18 (7–35)	6.5 (4–50)	9%
Tolenaar *et al*. 2013^35^	1Type I endoleak; 1 RA occlusion	0		2	1tumor 1 M.I. (26 months)	90.9%	10.87 (m4–19.4)	4 (3–9.5)	0
Lgari *et al*. 2014^36^	1 pneumonia; 1Type II endoleak;	0		0		100%	11 (2–22)	N.D.	0
Banno *et al*. 2014^37^	1 arrhythmia; 1 COPD; 2 bowel ischemia; 1 colitis; 2 cerebral infarction; 8 wound complications; 3 intra-abdominal or retroperitoneal hemorrhage; 1 urinary tract infection; 2Type I; 2Type IIendoleaks; 7 RFI;1 dialysis;2 Renal infract	3	3 bowel ischemia M.O.F.	4	Not related to AAA	95.2% (12 months)	12 (0–48)	N.D.	28%

**N.D.** not documented; **M.I.** myocardial infarction; **COPD**, chronic obstructive pulmonary disease; **M.O.F.** multiple organ failure; **RFI**, renal function impairment; **ARF**, acute renal failure; **RF**, renal failure; **RA**, renal artery; **SMA**, superior mesenteric artery.

**Table 8:** F-EVAR cohort clinical outcomes.

Authors	MAE (major adverse events)	30-day mortality	Cause of death	Over-30-day mortality	Cause of death	Patency (6 months)	Follow-up (months)	Length of stay (days)	Secondary intervention rate
Lee *et al*. 2014^38^	2 MI; 1 stroke;	0		2	Not related to AAA	96%	6	4 (2–23)	13.30%
	3 Type IIand 1 Type III endoleaks;								
	1 RA occlusion								
Globalstar 2012^18^	8 MI; 5 cardiac failure; 7 arrhythmia; 8 pneumonia;	2	Not related to AAA	11	Not related to AAA	98%	6	9 (1–100)	10% (12 months)
	3 COPD; 5 GI ischemia;6 sepsis or septicemia;								
	9 wound complications; 3 TIA; 5 spinal ischemia; 3 lower limb ischemia;								
	17 Type I;22 Type II;5 Type III endoleaks;								
	2 RA perforation;1 RA stenosis; 4 RFI; 1 ARF; 11 RF;1 RA occlusion;3 Renal branch Bleeding;								
Liao *et al*. 2014^39^	1 splenic embolization;	0		2	1 C.O.P.D. + heart failure 1 bowel ischemia + M.O.F.	N.D.	6.1 (2.7–8.3)	3 (1–9)	0
	2 Type IIendoleaks; 1 renal hematoma;								
Dijkstra *et al*. 2014^40^	1 compartment syndrome left lower leg; 1 rupture of common iliac artery; 1 occluded SMA; 1 cutaneous bleeding; 1 hemorrhagic CVA;	1	M.O.F.	1	1 stroke (6 months)	96% (1 month)	11 (1–29)	N.D.	0
	5 Type I, 12 Type II and 4 Type III endoleaks;								
	1 RFI; 1 RA occlusion								
Donas *et al*. 2012^31^	1 occluded SMA;	0		N.D.		97.7%	13.2 ± 4.2	3.5 ± 1.1	3.4%
	3 Type I and 7 Type II endoleaks;								
	1 LRA occlusion								
Suominen *et al*. 2013^32^	3 wound infection; 1 MI; 1 occluded common iliac artery;	2	1 pneumonia 1 MI	3	1 stroke (51 months)	N.D.	22 (1–46)	N.D.	10% (12 month)
	1 Type II endoleak;				1 gastrointestinal bleeding (12 months)				
	1 RFI; 1 stent twist				1 tumor (41 months)				
Greenberg *et al*. 2009^41^	2 arrhythmia; 7 transfusions; 1 low extremite embolus;	0		2	not related to AAA (677 days)	89%	24	3.7 (1–8)	17%
	1 supplemental O2 ; 1 paralytic ileus; 1 wound infection 3 CHF; 1 arrhythmia; 1 pneumonia; 2 incisional hernia;				1 MI (754 days)	51/57			
	1 Type I, 1 Type II and 1 Type III endoleaks;								
	2 RFI; 4 RA stenosis; 2 RA occlusion								
Tambyraja *et al*. 2011^42^	3 Iliac limb stenosis/occlusion; 1 SMA occlusion;	0		4	1 stroke (22 months)	N.D.	20 (7–62)	3 (1–12)	38%
	2 Type I, 5 Type II and 2 Type III endoleaks;				1 M.O.F. (18 months)				
	1 RA perforation; 9 RA stenosis; 2 RA occlusion; 3 stent migration				1 pneumonia (15 months)				
					1 renal failure. (18 months)				
Oderich *et al*. 2014^43^	3 bowel obstruction; 1 bowel obstruction; 1 stroke; 1 MI; 3 CHF; 2 cardiac ischemia;	1	Bowel ischemia (related to AAA)	4	1 M.O.F.	95%	37 (3–65)	3.3 ± 2.1	22%
	1 Type I and 16 Type IIendoleaks;				2 M.I.				
	4 RA occlusion;12 RA stenosis; 8 RFI; 3 Renal failure				2 unknown cause				

**N.D.** not documented; **M.I.** myocardial infarction; **COPD**, chronic obstructive pulmonary disease; **M.O.F.** multiple organ failure; **RFI**, renal function impairment; **ARF**, acute renal failure; **RF**, renal failure; **RA**, renal artery; **SMA**, superior mesenteric artery.

**Table 9:** Preoperative patient demographics and main outcomes in F-EVAR and CH-EVAR cohorts.

	F-EVAR	CH-EVAR	*P* value
*Preoperative*			
Age	74 (47–86)	75 (59–88)	
Aneurysm diameter	64 (47–112)	64.5 (33–110)	
Length of aneurysm neck	6.7 ± 3.6(0–14.4)	2.3 ± 4.3 (0–10)	
*Outcomes*			
Operative time (min)	261 (80–554)	178 (75–810)	
Fluoroscopy time (min)	64 (5–223)	54.6 (15–290)	
Contrast dose (ml)	166 (90–465)	146 (45–465)	
Estimated blood loss (ml)	534 (50–7000)	332 (30–2204)	
Technique success rate	98.8%	97.4%	0.15
30-day mortality	6 (1.1%)	8 (3.8%)	0.02
Over-30-day mortality	29 (5.35%)	15 (9.5%)	0.01
All-cause mortality	35 (6.46%)	21 (13.3%)	0.0002
Patency	95.9%	97%	0.34
Follow-up (month)	12.8 (1–65)	14.7 (0–46)	
Length of stay (day)	7 (1–100)	4.4 (2–50)	
Secondary intervention rate	58 (10.7%)	17 (9.5%)	0.98
